# Transactions of the Buffalo Obstetrical Society

**Published:** 1886-05

**Authors:** 


### Society Reports.

### TRANSACTIONS OF THE BUFFALO OBSTETRICAL SOCIETY.

*Regular Meeting, January 25, 1886.*

The President, Dr. W. W. Potter, in the chair; Dr. W. H. Thornton, Secretary.

The election of officers for 1886 resulted as follows:

President, DR. W. W. POTTER; Vice-President, DR. R. L. BANTA; Secretary and Treasurer, DR. W. B. HAWKINS.

DR. C. R. JEWETT read a paper on "The Management of the Third Stage of Labor."

The discussion of the paper was postponed, to enable the Society to join the "Medical Union" in its annual dinner.

---

The regular meeting of February 23, 1886, was adjourned to March 1st, on account of the commencement exercises of the Medical Department of the University of Buffalo, and on the latter date Dr. W. W. Potter read a paper entitled, "Some Observations on the Therapeutic Uses of the Uterine Sound."

DR. J. W. KEENE occupied the chair, and the paper was discussed by Drs. Lothrop, Baker, Van Peyma, Clark, Hawkins, Keene, and the author.

---

*Regular Meeting, March 23, 1886.*

The President, Dr. W. W. Potter, in the chair; Dr. W. B. Hawkins, Secretary.

DR. P. W. VAN PEYMA read a paper entitled, "Some Points in the Use of the Obstetrical Forceps."

After referring to the dread with which the text-books had inspired him, as a student, to regard the forceps, and a somewhat humorous recital of his first experience with the instrument, he stated that the main object of the paper was to emphasize his strong belief in the usefulness and harmlessness of the forceps, when employed with the most ordinary prudence and common sense, in many cases where Nature may be capable to effect a delivery unaided, but after hours of additional suffering, and with increased danger to both mother and child. He then stated that this opinion was based upon an experience extending through a practice of more than ten years, and embracing an attendance upon a total of more than 650 cases of obstetrics. Of these, the larger majority were cases presenting some abnormal conditions, and fully 300 were widwife cases.

For years, the number of midwife and other abnormal cases had far exceeded the number of those where the writer was in attendance from the beginning of labor, or where the progress was normal. In fact, the cases not originally attended by midwives were, with very few exceptions, either primiparæ, or women who, for some reason—perhaps previous experience of difficult labor—desired the attendance of a physician throughout.

The 300 midwife cases, with others occuring in his own practice and that of other physicians, included at least 150 forceps deliveries. The abnormal conditions existing in these cases, and requiring the use of the forceps, were complete or partial cessation of labor pains, rigid os uteri, malpositions, convulsions, face presentations, after-coming head, prolapsus of the funis, deformed pelvis, monstrosities, and placentæ previæ.

It should be remembered, said he, that some midwives attended, yearly, as many as 200 to 250 cases; and taking the practice of half-a-dozen of these midwives for ten years, would give an aggregate of from 12,000 to 15,000 births. A physician, therefore, who was the exclusive consultant for those six midwives, could claim to have seen the abnormal cases of at least 10,000 confinements. He strongly emphasized the difference between this and an ordinary obstetric practice, since it was often overlooked and rarely appreciated.

In connection with the well-known rules for applying the forceps, he would only call attention to a very important and much neglected injunction, viz., to be sure that the blades of the instrument passed within the rim of the uterine mouth. The point was then made that the application of the forceps does not imply the employment of force. The large majority of forceps deliveries were stated to be in consequence of a want of the normal vis-a-tergo, and the writer asked, "Why should we hurry in these cases?" The forceps has furnished the compliment of force, sufficient traction having been added to make the combined forces equal to the normal. The initiatory traction having started the head, it is frequently unnecessary to pursue it further, the normal uterine contractions having thereby been set to work to some purpose. Yet, even in such cases, he considered it unobjectionable to allow the forceps to remain *in situ* until the end of the second stage, and that earlier removal was useless, since the instrument might be required at a later period.

The commonly accepted view that there was no danger to the child during the first stage was believed to be erroneous, for he had seen the head engage and become exceedingly compressed during this stage, the uterine walls preceding the head, and becoming likewise injured. An instance was cited in which the child was alive two hours before the termination of labor, and yet, though the second stage lasted but an hour, the child was born dead. In the case of this woman the first stage usually lasted a week, but, by artificial dilatation of the os and the application of the forceps, he had been able, subsequently, to deliver her twice of living and healthy offspring, besides saving her days of suffering.

In cases of natural head presentation, with too early rupture of the membranes and draining away of the amniotic fluid, he believed the os should be dilated and the forceps applied, delay being dangerous through pressure of the uterine walls upon the child and cord. But in all such instances, deliberation and patience were necessary, and sufficient time should be allowed for the head to become elongated by gradual pressure. The amount of traction force was, in some cases, enormous; though these were rare exceptions. Slipping of the forceps should be exceedingly rare. Bringing the handles forward too rapidly was believed to be a frequent cause of slipping, and should therefore be avoided.

A motion from side to side, and from before backward and the reverse, during traction, was, within certain limitations, considered useful, notwithstanding the objection in principle that any lateral swinging must increase, temporarily, the transverse diameter. This increase, however, he declared to be absolutely insignificant, while, by means of leverage, a far less amount of direct traction was required, thus making the delivery not alone much easier to the accoucheur, but also diminishing the pressure upon the maternal soft parts, and the scalp of the child.

In cases where the forceps could not be made to lock easily, he advised tenative traction until the head came down somewhat, or at least changed its position, when the instrument could be regularly applied; also, in cases where a tendency to twist was apparent, locking the forceps having been already accomplished.

Regulating the line of expulsion by positioning the pelvis of the mother during the successive stages of delivery, was a manœuvre which he thought would materially assist the operator, particularly in cases of projecting sacral promontory. The recognition of slight deformities and the consequent need of the forceps was difficult; and the simple fact that labor was retarded in an otherwise apparently normal case, he considered a safe guide to its use. In face presentations, where the head did not fully engage, and the uterus was not too firmly contracted, he believed version to be much the more preferable operation, but stated that, with the woman in the knee-chest position, the vertex might sometimes be brought down, and the forceps applied to hold it in its new position.

Regarding the treatment of the after-coming head in either anterior or posterior positions, he strongly advised the use ot the forceps if there was more than two minutes' delay in delivery. An exception to this rule was illustrated by a case in which twisting of the neck had taken place, the chin catching on the pubes; but as soon as the chin had been rotated posteriorly by the finger introduced into the vagina, the head was spontaneously delivered. Finally, in cases of prolapsus of the funis, the head not having engaged, he had found it very useful, after repositing, with the woman in the knee-chest position, to apply the forceps and retain the head, until a few pains had forced it into the grasp of the cervix.

DISCUSSION.

Dr. Milan Baker, in opening the discussion, said that while the forceps occupied a very high place in the domain of obstetrics, he felt like uttering a few words of caution and warning concerning its use. He thought it was often resorted to when simpler expedients would suffice, and counseled calm deliberation and judgment in every case where they seemed indicated. Thought also, the traction force used should be as little as possible, and the greatest care taken to exercise it in the proper direction.

Dr. Charles G. Stockton thought the paper one of great value, based as it was on personal experiences; said he could best give his views of the subject by relating two cases which would illustrate the points he desired to make. The first case was in the person of a healthy, large, muscular woman, a primipara, to whom he was called one night after she had been in labor several hours. After waiting some. time and noting no progress, he applied the forceps, and even used much force, but was wholly unable to cause the head to advance any. Dr. Tremaine was then called and expended all his available strength with a like result; when, finally, Dr. Lothrop came, but whose heroic efforts were rewarded with no better results than those of his muscular predecessors. Notwithstanding all this, at daybreak a large child was naturally delivered—dead. The second case was that of a delicate woman who had made slow but gradual progress. Here, as the force seemed sufficient, he decided to wait, and after having been in labor twenty-four hours, the woman was delivered naturally of a dead child, weighing sixteen pounds. The first case did well after her confinement, but the second had a vesico-vaginal fistula, which may, however, have been caused by a calculus, as quite extensive phosphatic deposits were afterward found in the bladder. He used his best judgment in both cases at the time, and thought the question when to use the forceps and when not often a very hard one to decide.

Dr. George E. Fell thought highly of the forceps, and spoke of a very favorable experience with the instrument. He believed, however, that it was too often used carelessly and recklessly, and mentioned a case seen at the hospital where the instrument was brutally used, no care having been taken to apply the traction force in the axis of the pelvic strait, the child having been literally dragged directly through the perineum.

Dr. W. H. Thornton said he had never used the forceps, but believed it could be employed in many cases to the great benefit of both mother and child.

Dr. Thos. Lothrop stated that he applied the forceps now-a-days much less frequently than ten years ago, but he did not wish to be understood as in any way depreciating the judicious use of the instrument, for he regarded it as the most important obstetric aid which we possessed. He had, however, been unsuccessful with it in cases complicated by prolapsed funis.

Dr. C. C. Frederick asked the author if he would dilate and apply the forceps in the first stage of labor under any conditions.

Dr. J. W. Keene said that it had not been necessary, in his experience, to use the forceps with great frequency. He also thought the application of the instrument to the after-coming head not as easy or as devoid of danger as it was often represented to be.

Dr. F. H. Potter, referring to the adoption of the genu-pectoral posture for repositing prolapsed funis, said that it sometimes entirely suspended labor pains, and always markedly lessened their force for a time, upon which fact greatly depended the possibility of replacing the cord.

Dr. R. L. Banta was greatly interested in the subject, and related, in a humorous way, his first experience in the use of the forceps. He had not obtained much success with the forceps in face presentations, and feared he had done more harm than good with it in those cases; thought it a very difficult matter to apply the instrument to an after-coming head, and even when accomplished, the child would almost invariably be born dead. He also believed it well nigh impossible, in cases of funis prolapse, to save the child with the forceps; and further expressed the opinion that the forceps should never be used in the first stage of labor.

Dr. W. B. Hawkins had been obliged to use the forceps in but three instances, and in each its use was attended with most beneficial results to both mother and child. He felt quite sure that in several cases of tedious labor, due apparently to uterine inertia, he had been able to avoid resorting to the forceps, by the application to the mother's abdomen of large, hot poultices, which, by their heat, very soon stimulated the uterus to firm and regular contractions.

The President remarked that he quite agreed with Dr. Banta in considering it next to impossible to save the child by the use of the forceps in cases of prolapse of the funis; so, too, in all cases of after-coming head. In all such junctures, he preferred to deliver by firm and resolute supra-pubic pressure. The medical world might, he said, be divided with regard to the use of the forceps into two general classes: First, those who always carry the instrument with them when going to attend cases of labor; and, second, those who never take it along, but send for it when needed. The first class make frequent use of it under any and every pretext, while the second rarely invoke its aid, and then only after Nature's powers have been sorely tested and found wanting; or, in other words, under the exigencies of dire necessity. A conservative obstetrician would approve the course of the latter; but it sometimes happened that sudden emergencies arose, when a few moments lost were to miss a golden opportunity to save life. It were better, on the whole, to always be prepared for any contingency, and then act with due deliberation in the use of the forceps. In a doubtful case whether or not to apply it required much experience and a calm judgment to decide. Where uterine contraction was very strong, and dangerous from its very strength, he believed its application, by hastening delivery, did much good.

The vital points essential to a successful performance of this obstetric operation were, in his opinion, first, the perfection, as near as might be, of one's own self in the application of the instrument; second, a perfected judgment and perception of the needs of each case; and, lastly, to carefully study and estimate the amount of force required.

Regarding the pendulum movement in using the forceps, he would only employ it with extreme caution, and after failure with direct traction. He had been able, on a few occasions, to apply the forceps in the Sims' position, after failure in the ordinary way.

Dr. Van Peyma, in closing the discussion, said that often simply introducing the blades, or even but one of them, would rouse the pains, and labor would be completed without the employment of any traction force whatever. On the other hand, he had seen cases where he was obliged to employ all possible force for two hours. In the first stage of labor, he would use forceps after twenty-four hours' delay, especially if the amniotic fluid was escaping; applying the blades gently at such times, or, possibly, only one blade, making gentle, oscillating movements, believing that this procedure would save both mothers and children many times. It was true, he said, that the knee-chest position would sometimes temporarily cause cessation of the pains, but that he had often seen hard pains continue during the whole procedure. In the delivery of the after-coming head, he believed firmly in the efficacy of the forceps, and in the possibility of saving the child by it if used soon enough, reiterating the statement made in the paper that the instrument should be applied if more than two minutes' delay occurred after the birth of the shoulders.

